# Supplementary material for: Proteomic Blood Profiles Obtained by Totally Blind Biological Clustering in Stable and Exacerbated COPD Patients
Source: Cells. 2024 May 17;13(10):866. doi: 10.3390/cells13100866 (PMC11119172; doi:10.3390/cells13100866)
Supplement: Supplementary file 1 [file cells-13-00866-s001.zip › cells-2956578-supplementary.pdf]

SUPPLEMENTARY MATERIAL

Proteomic Blood Profiles Obtained by Totally Blind Biological Clustering in Stable and Exacerbated COPD Patients

Cesar Jessé Enríquez-Rodríguez [1,2,3,4], Sergi Pascual-Guardia [1,2,3,4], Carme Casadevall [1,2,3,4], Oswaldo Antonio Caguana-Vélez [1,2,3,4], Diego Rodríguez-Chiaradia [1,2,3,4], Esther Barreiro [1,2,3,4] and Joaquim Gea [1,2,3,4,*]

1 Respiratory Medicine Department, Hospital del Mar—IMIM, 08003 Barcelona, Spain
2 MELIS Department, Universitat Pompeu Fabra, 08003 Barcelona, Spain
3 CIBERES, ISCiii, 08003 Barcelona, Spain
4 BRN, 08003 Barcelona, Spain
* Correspondence: quim.gea@upf.edu

Post-hoc analysis using Immune-based multiplex

Since our blind proteomic analysis may have led to the loss of identification of proteins/peptides that are relevant in the immune response but have relatively low blood levels, we performed a post-hoc complementary approach, driven by hypothesis. For this, we explored those proteins considered as the most probable markers of COPD and/or AECOPD using immunology-based multiplex in the same blood samples.

Methods

Plasma concentrations of a panel of soluble markers were examined by using three commercial multiplex bead-based immunoassays (Bio-Rad, Hercules, CA, USA), following the manufacturer's instructions. We included a total of 55 markers (cytokines, chemokines, growth factors and acute phase proteins), selected according to standard literature knowledge and an earlier specific text mining [1].

Furthermore, the same steps used for the analysis of the results obtained by LC-MS/MS were used here (machine-learning unsupervised cluster generation, proteome differential analysis between clusters, analysis of differences between general clinical characteristics between the obtained clusters, and the final confrontation of clusters with different clinical groups).

Results

By using this complementary approach, concentration values were obtained in 9 acute phase proteins and 45 cytokines/chemokines/growth factors (Table S1). Peptides from the constant region of the Ig heavy chain were assumed to represent Ig isotypes. Then, 11 clusters were generated using the above-mentioned method for multiplex results and 11 more for the addition of the latter technique to those from LC-MS/MS (Table S2). As in the case of LC-MS/MS, the cluster analysis using multiplex was unable to appropriately segregate stable COPD patients from healthy individuals. Although multiplex results were slightly better in terms of specificity than those obtained with LC-MS/MS the sensitivity remained extremely low, resulting in a poor-to-moderate accuracy [2]. Moreover, the combination of both proteomic techniques to generate clusters adds no further information (Tables S4 and S5). The same procedure used for the LC-MS/MS was also performed in this case to identify AECOPD, but the results were worse, and the combination of both proteomic techniques did not significantly improve the accuracy of the prediction (Tables S6 and S7).

Tables S1–S3
See in the attached Supplementary EXCEL File

Table S4. Main clinical characteristics in each Kmeans-2 cluster found by proteomics, and confrontation of these clusters with the distribution of actual COPD and Control groups.

	LC-MS/MS		Immuno-based Multiplex		Both Techniques together	
**Clusters**	**A**	**B**	**A**	**B**	**A**	**B**
**Individuals, n**	24	10	21	13	16	18
**General characteristics**						
**Age, yr.**	64±9	67±10	66±7	64±12	63±9	67±9
**Males, n (% in the cluster)**	12 (50)	8 (80)	13 (62)	7 (54)	9 (56)	11 (61)
**BMI, kg/m²**	25.7±6.2	23.1±4.8	26.0±6.4	23.2±4.5	22.6±3.6	27.1±6.7*
**Group**						
**CONTROL, n (% in the cluster)**	8 (33)	2 (20)	5 (24)	5 (38)	3 (19)	7 (39)
**SCOPD, n (% in the cluster)**	16 (67)	8 (80)	16 (76)	8 (62)	13 (81)	11 (61)

*Values are expressed as mean± SD, or percentage. Significance: *, p < 0.05 Cluster B compared with Cluster A;* ***Abbreviations***: *BMI, body mass index; SCOPD, stable COPD.*

Table S5. Clustering outcomes for COPD diagnosis.

Assay	N of clusters	SP	SE	PPV	PNV	ACC	MCC	Raw p-value	Bonferroni
**LC-MS/MS**	2	20 (16)	67 (19)	67 (19)	20 (16)	53 (20)	-0.13	0.68	1.0
**Immune-based Multiplex**	2	50 (20)	67 (19)	76 (17)	39 (20)	62 (19)	0.16	0.45	1.0
**Both**	2	70 (18)	54 (20)	81 (16)	39 (20)	59 (20)	0.22	0.27	0.8

Values are expressed as percentage (CI95). Cross-validation Fisher and Bonferroni p-values are included. ***Abbreviations:*** *SP, Specificity; SE, Sensitivity; PPV, Predictive Positive Value; PNV, Predictive Negative Value; ACC, Accuracy; MCC, Matthew's correlation coefficient.*

Table S6. Main clinical characteristics in each Kmeans-2 cluster found with both laboratory techniques and confrontation of these clusters with the distribution of exacerbated and stable COPD patients.

	LC-MS/MS		Immuno-based Multiplex		Both techniques together	
**Cluster**	**A**	**B**	**A**	**B**	**A**	**B**
**Individuals, n**	(13)	(21)	(15)	(19)	(15)	(19)
**General characteristics**						
**Age, yr**	65.9±8.3	64.2±9.0	63.8±7.7	65.7±9.4	65.2±8.3	64.6±9.1
**Males, n (% in the cluster)**	6 (46)	13 (62)	7 (47)	12 (63)	8 (53)	11 (58)
**BMI, kg/m²**	29.3±7.4	23.6±4.8*	26.8±7.3	25.0±5.9	29.2±7.3	23.1±4.2**
**COPD group**						
**SCOPD, n (% in the cluster)**	5 (39)	19 (91)**	12 (80)	12 (63)	7 (47)	17 (90)**
**AECOPD, n (% in the cluster)**	8 (61)	2 (9)**	3 (20)	7 (37)	8 (53)	2 (10)**

*Values are expressed as mean± SD, or percentage. Significance: *, p<0.05 B compared to A;* ***Abbreviations****: AECOPD, acute exacerbated COPD; SCOPD, stable COPD; BMI, body mass index.*

Table S7. Best clustering outcomes for identification of exacerbations.

Assay	N of clusters	SP	SE	PPV	PNV	ACC	MCC	Raw p-value	Bonferroni
**LC-MS/MS**	**2**	**79 (25)**	**80 (25)**	**62 (30)**	**91 (18)**	**79 (25)**	**0.55**	**< 0.01**	**< 0.01**
**Immune-based Multiplex**	**2**	50 (31)	70 (28)	37 (30)	80 (25)	56(31)	0.18	0.45	1.00
**Both**	**2**	**71 (28)**	**80 (25)**	**53 (31)**	**90 (19)**	**74 (27)**	**0.47**	**<0.01**	**0.03**

Values are expressed as percentage (CI95). Cross-validation Fisher p-value and Bonferroni correction value are shown. ***Abbreviations:*** *SP, Specificity; SE, Sensitivity; PPV, Predictive Positive Value; PNV, Predictive Negative Value; ACC, Accuracy; MCC, Matthew's correlation coefficient.*

References

1. Grosdidier, S.; Ferrer, A.; Faner, R.; Piñero, J.; Roca, J.; Cosío, B.; Agustí, A.; Gea, J.; Sanz, F.; Furlong, L.I. Network Medicine Analysis of COPD Multimorbidities. *Respir. Res.* **2014**, *15*, 111, doi:10.1186/s12931-014-0111-4.

2. Kelleher, J.D.; Mac Namee, B.; D'Arcy, A. *Fundamentals of Machine Learning for Predictive Data Analytics: Algorithms, Worked Examples, and Case Studies*; The MIT Press: Cambridge, Massachusetts, **2015**; ISBN 978-0-262-02944-5.
